# Bridging the care gap: patients’ needs and experiences regarding shared decision-making in radiotherapy

**DOI:** 10.1016/j.ctro.2024.100897

**Published:** 2024-11-24

**Authors:** A.R. van Hienen, C.J.W. Offermann, L.J. Boersma, M.J.G. Jacobs, R.R.R. Fijten

**Affiliations:** a Department of Radiation Oncology (MAASTRO), GROW Research Institute for Oncology and Reproduction, Maastricht University Medical Centre, Maastricht, Dr Tanslaan 12, 6229 ET, the Netherlands; b Maastro Clinic, Research Affairs department, Maastricht, Dr Tanslaan 12, 6229 ET, the Netherlands; c Department of Management, Tilburg School of Economics and Management, Tilburg University, Tilburg, Warandelaan 2, 5037 AB, the Netherlands

**Keywords:** Shared Decision Making, Patient Preference, Physicians, Neoplasms, Educational Status, Patient Reported Outcome Measures, Referral and Consultation, Tertiary Care Centers

## Abstract

**Background and purpose:**

Shared decision-making (SDM), a collaborative process in which patients and physicians jointly determine further treatment, has been associated with numerous positive effects. However, its implementation into routine clinical practice faces challenges. In radiotherapy (RT) it may have additional challenges, since patients are referred from another oncologist, often “to undergo RT”. The aim of this study is to understand patients’ preferences and experiences regarding SDM at an academic RT clinic, and to identify targets for SDM implementation in RT.

**Materials and methods:**

We adapted an earlier survey sent out by the Dutch Cancer Patient Organizations Federation to fit the RT setting. The survey was distributed via letters and social media to (former) patients who had their intake between 2020 and 2022.

**Results:**

1799 participants completed the survey, of whom 88,3% mentioned to always or often prefer SDM. 23,1% of participants reported experiencing a choice, and 50% of these participants experienced multiple options. The most commons reason for preferring SDM was bodily autonomy (n = 1114) and against SDM was wanting to decide themselves instead (n = 11). Participants with a higher educational attainment were more likely to prefer and experience SDM. Older participants were more likely to experience multiple options.

**Conclusion:**

Our findings reaffirm that most cancer patients prefer SDM, and extend these findings to RT. However, we found a large gap between patients’ desire for SDM, and the SDM experienced in our RT institute. SDM implementation strategies are needed and should focus on overcoming RT-specific and patient-reported barriers and opportunities.

## Highlights

• 88,3% of RT patients prefers shared decision-making (SDM)
• 23,1% of patients did not experience a choice during their RT intake.
• Highly educated patients were more likely to prefer and experience SDM.

## Introduction

Shared decision making (SDM) can optimize cancer treatment by having patients and oncologists decide on treatment together, considering the best available evidence and the patient’s preferences [1]. SDM can foster patients’ rights to self-management and bodily autonomy, and can improve treatment adherence, satisfaction, and perceived quality of life [2], [3], [4], [5]. Yet, SDM is still underutilized in oncology [6], [7], [8], [9], despite the multitude of preference-sensitive treatment decisions [10], the often sufficient time window for SDM between diagnosis and treatment [11], [12], [13], and cancer patients’ preference for an active or shared role in medical decision-making [14]. To illustrate, in a survey of the Dutch Cancer Patient Federation (n = 3785), 88,1% of Dutch cancer patients reported to prefer an active or shared decisional role, while only 58% of patients reported that multiple treatment options were offered. Discussion of patient values and future aspirations occurred in less than half of these cases [15], suggesting unmet needs for SDM in oncology in the Netherlands.

While Elwyn’s four-step model of SDM is useful for implementation in any clinical setting [16], implementation strategies are most effective when they are aligned with their context-specific barriers and facilitators [17], [18]. Barriers to SDM in general oncology include limited continuity of care, oncologists’ overestimation of their own SDM use and time requirements, and patients’ hesitance to ask questions [11], [19], [20], [21], [22]. However, these studies failed to examine the settings of oncology specialisms like radiotherapy (RT), which may have additional facilitators and barriers. Literature suggests that radiation oncologists were more likely to prefer shared or patient-led decision-making [23], and that available RT regimens are often numerous, complex and preference-sensitive [24], but whether these factors facilitated SDM was not assessed. Unlike systemic treatments, patients always need to be referred to a radiation oncologist by their treating physician to access RT. This treating physician may already provide initial information about RT. A survey of 403 US breast cancer surgeons indicated limited RT knowledge, potentially limiting the quality of information patients receive from their treating physician about RT [25], which could inhibit their ability to partake in SDM.

Until now, no studies have measured patients’ SDM preference and experience in RT clinics, and due to the specific characteristics of the RT setting, knowledge about patients’ SDM preference and experience from general oncology may not be extrapolated without verification. This knowledge is important, as it indicates to what extent patients’ SDM preferences in RT match with their experiences, and thus, to what extent implementation strategies addressing the facilitators and barriers in RT are required. Therefore, the aim of this study is to assess patients’ preference and experience with SDM in an academic RT clinic in the Netherlands, and to identify targets for SDM implementation strategies in RT.

## Materials and methods

### Study design

This study used a cross-sectional survey for patients and was approved by the Institutional Review Board.

### Materials

**Fig. 1:**
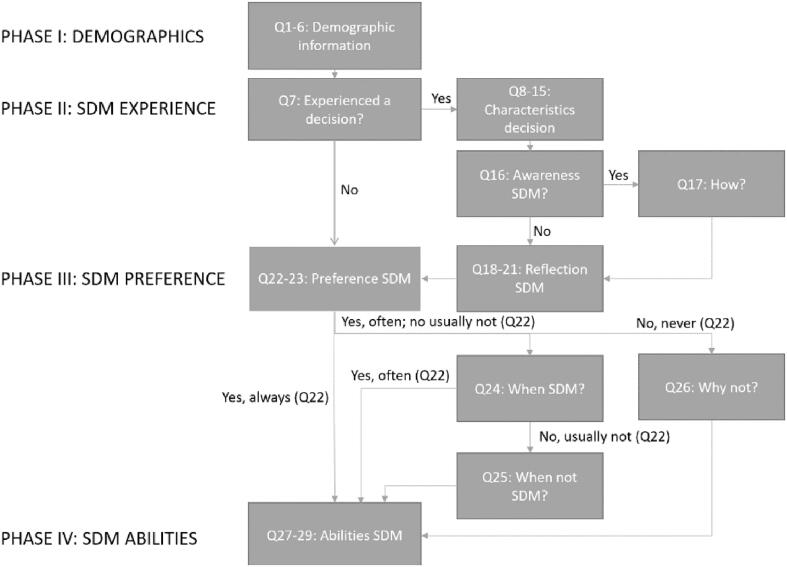
Flowchart describing the items patients needed to fill in based on previous responses. sdm = shared decision-making, Q = question.

A Dutch language survey was developed, consisting of 29 items divided over four phases: 1) demographic characteristics, including type of cancer, 2) patients’ experiences with decision-making at the clinic, 3) patients’ desired SDM at the clinic and 4), patients’ perceived ability to take part in SDM. Questions included “Did you experience, with the radiation oncologist, making a choice about a treatment?” (Q7) and “Do you want to engage in shared decision-making with your healthcare provider?” (Q22), and answering options were multiple-choice. The survey was derived of the survey administered by the Dutch Cancer Patient Federation and reviewed by experts to ensure content and face validity [15]. Adaptations were made to limit the scope to RT, items no longer relevant were omitted or adapted, and items on motivations and RT-specific circumstances were added. The items participants needed to respond to were partially guided by their earlier answers to limit participant burden (Fig. 1), for example, only patients who indicated having experienced a decision were asked what this decision was about. All materials, scripts and a curated dataset are freely accessible here: https://doi.org/10.17605/OSF.IO/GW2NB.

The survey was digitally accessible between September 2022 and February 2023. An English version is available in the supplemental materials. No specific efforts were made to minimize human errors in data entry, however, selection of the right answer was not considered highly difficult. The survey was designed to be anonymous, as no data on participants’ name and address were collected.

### Sample characteristics

Participants were included if they had an intake consultation with a radiation oncologist at the RT centre in Maastricht or Venlo (The Netherlands) between 2020 and September 2022. 7158 (former) patients of the RT centre were sent a personalised letter with a QR code and URL to the digital survey. Patients could also request a paper survey to be mailed to their homes.

Within the Dutch educational system, tertiary education is roughly divided in three categories, medium vocational training, higher vocational education and academic education. Medium vocational training equips students with the knowledge and skills required for more practical jobs like kitchen staff, teacher assistant or office support specialist. Higher vocational education and academic education both fall under higher education, but higher vocational education is profession oriented, whereas academic education is more research oriented.

### Statistical analyses

The analyses were performed using R version 4.3.1 [26] and RStudio version 2023.12.1, build 402, using the base R statistics when possible, “rcompanion” [27] to calculate Cramér’s V [28], “ggplot2” [29] to create graphs and “tidyverse” [30] for data processing.

A significance level of p-value < 0,05 was used for all analyses. Cancers with less than 5 respondents were coded as “other cancers”. Categorical variables were expressed using absolute numbers and percentages, calculated with the number of entries for the item as denominator. The variable age was grouped into quartiles. Pearson’s chi-square test was used to test for independence.

## Results

In total, 1799 patients filled in the survey. 1788 patients responded using the letter sent to all patients having an intake 2020–2022 (n = 7158), leading to a response rate of 25,0%. Additionally, 11 patients accessed the survey via social media. Reasons for non-response were not recorded. The sample had a mean age of 67,6 (SD = 10,9) years and consisted of 52,8% men. The most common educational levels were medium vocational training (33,5%) and higher vocational education (25,7%). The most common cancers were breast (28,2%) and prostate cancer (25,2%). The overview is provided in Table 1.

**Table 1:** Overview of patient characteristics, including demographic characteristics, treatment year and disease. iqr = interquartile range. For treatment years and disease, participants were able to select multiple options, resulting in more observations than participants. 201 patients received treatment in > 1 year. 41 patients were treated for > 1 disease. Percentages may not total 100 due to rounding.

	N	%
Gender (n = 1799)		
Woman	847	47,1%
Man	950	52,8%
Non-binary	0	0,0%
Do not want to share	2	0,1%
Age in years (n = 1799)	Mean = 67,6; IQR = 13	
Educational level (n = 1799)		
Primary education	132	7,3%
Secondary education	429	23,8%
Medium vocational training	602	33,5%
Higher vocational education	462	25,7%
Academic education	134	7,4%
Other/Do not want to share	40	2,2%
Treatment year (n = 2000)		
2020	552	27,6%
2021	751	37,6%
2022	697	34,9%
Disease (n = 1840)		
Breast cancer	520	28,3%
Prostate cancer	463	25,2%
Lung cancer	197	10,7%
Head-neck cancer	174	9,4%
Gastrointestinal cancer	162	8,8%
Haematological cancer	76	4,1%
Neurological cancer	63	3,4%
Gynaecological cancer	52	2,8%
Bladder cancer	35	1,9%
Skin cancer	25	1,4%
Renal cancer	8	0,4%
Other cancer	51	2,8%
Other non-cancer	14	0,8%

**Fig. 2:**
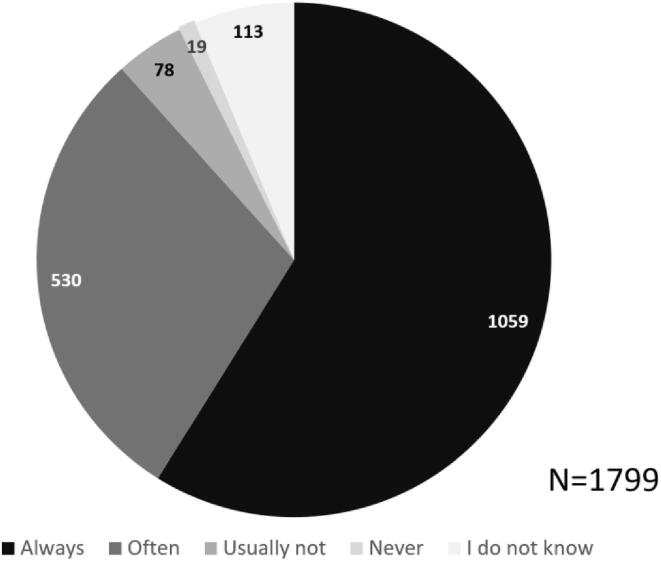

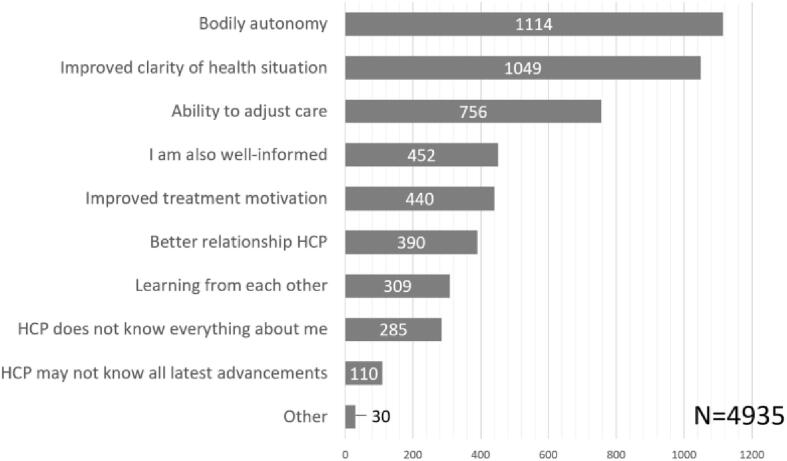

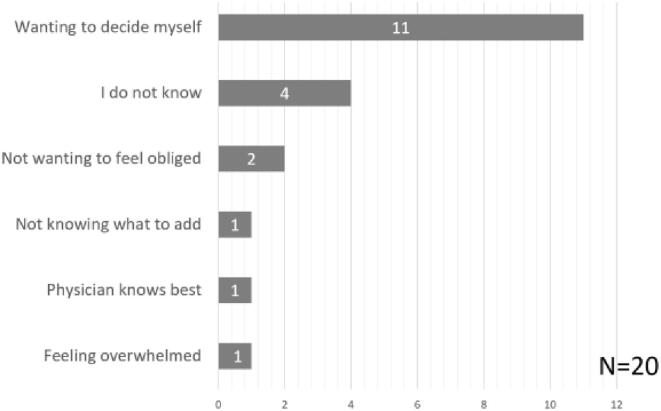
A. SDM preference. b. reasons for SDM preference. HCP = healthcare provider. c. reasons against SDM preference.

88,3% of participants preferred SDM, always (58,9%, n = 1059) or often (29,5%, n = 530). Most participants stated ownership of their body and improving clarity on their health situation as reasons *for* SDM, whereas preferring active decision-making was most prominent reason *against* SDM (Fig. 2).

Patients who often preferred SDM (n = 530) and who usually did not prefer SDM (n = 78), expressed finding SDM most suitable for decisions about life-threatening diseases or problems (n = 400) and when making care agreements (n = 340). Patients who expressed to usually not prefer SDM, most commonly expressed that they did not want SDM in cases of emergency (n = 48) and life-threatening diseases (n = 30). The majority of participants expressed always (20,1%) or often (54,1%) feeling able to contribute to conversations with their healthcare provider (Fig. 3a). As participants could select multiple options, 5692 responses were recorded with suggestions supporting patients to partake in SDM, of which listing pros and cons of treatment options (n = 1097) and the advice of the healthcare provider (n = 716) were selected most often (Fig. 3b).

**Fig. 3a:**
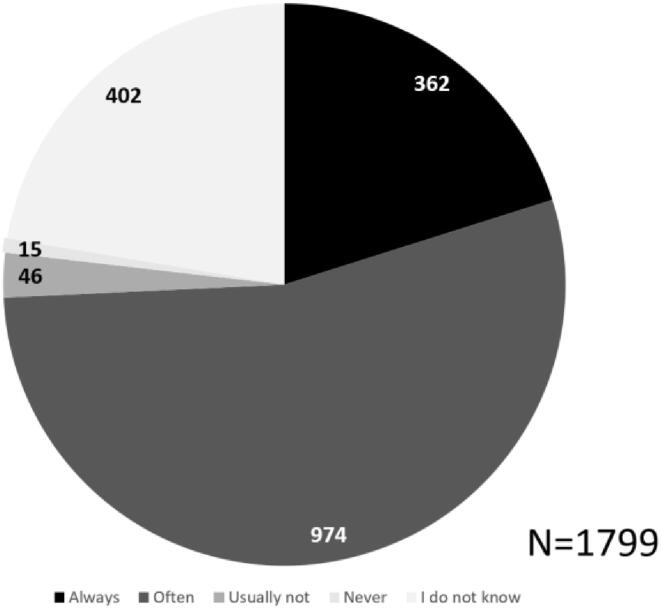
Perceived sdm ability.

**Fig. 3b:**
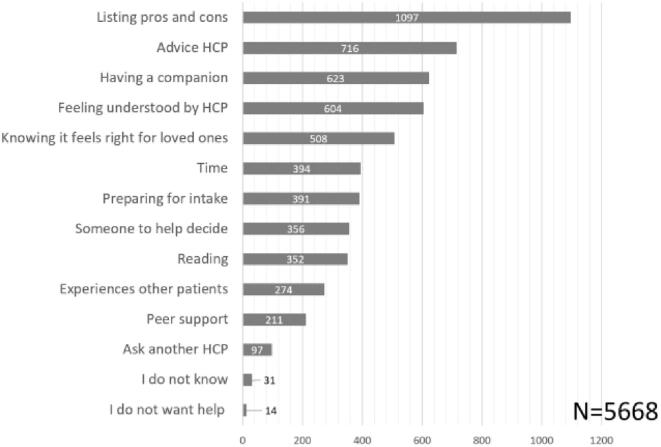
Strategies to be better equipped for sdm. HCP = healthcare provider. Participants could select multiple strategies to feel better equipped for SDM, resulting in a larger sample size.

70,9% (n = 1276) reported that they did not experience that a decision had to be made during the consultation, either by themselves or by the physician; 23,1% reported (n = 416) that they did experience that a decision had to be made. Most experienced decisions were about treatment initiation, mainly whether to initiate RT vs. another treatment (n = 79) or no treatment (n = 151), or the RT dose or fractionation (n = 99). 11,6% (n = 208) of all participants was provided with multiple options, 10,0% (n = 180) with one option and 1,6% (n = 28) with no options.

Of the 416 patients who had experienced that there was a choice to be made, 70,7% was aware in advance of themselves being able to participate in medical decision-making alongside their physician. Explanations for being aware of the ability to participate in medical decision-making in advance included finding SDM self-explanatory (n = 148, 35,8%), being informed by a healthcare provider (n = 134, 32,37 %) and reading about SDM (n = 55, 13,3%). Most of these participants (n = 332, 73,1%) did not experience difficulties taking part in SDM. For those that did find it difficult to participate, reported challenges included finding the consequences of the decision impactful (n = 42), and preferring to get advice from the radiation oncologist instead (n = 17).

The 416 patients who had experienced that there was a choice to be made ranked their satisfaction with their radiation oncologist’s performance of the steps of SDM. 98,3% (n = 409) rated the explanation of the personal health situation at least satisfactory. Satisfaction with physician’s explanation of treatment benefits was 79,1% (n = 329), of drawbacks was 76,9% (n = 320) and of risks was 75,0% (n = 312). 97,8% (n = 407) was at least satisfied with value elicitation and 96,9% (n = 403) with the consideration of patient values in making the decision. 92,8% (n = 386) was satisfied with their level of involvement in decision-making and 89,7% (n = 373) with their treatment.

Subgroup analyses were conducted to understand whether between-group differences in SDM preferences and experiences were present. Participants with a higher educational level were slightly more likely to prefer SDM (Cramér’s V = 0,08455, P = 0,0001398), as presented in Table 2. A higher educational level was also positively associated with experiencing a decision (Cramér’s V = 0,1291, P = 3,846e-09). Associations of gender, disease, treatment year and age with experiencing a decision were not significant.

**Table 2:** Overview distribution and effect size of age, gender, educational level and disease on sdm preference, experience and number of treatment options.

	Preference (n = 1799)		Experience (n = 1799)		Options^a^ (n = 416)	
	Cramér’s V (p)	% positive (n)	Cramér’s V (p)	% SDM (n)	Cramér’s V (p)	% Multiple (n)
Age (n = 1799)	V = 0,05281 (p = 0,2446)		V = 0,05649 (p = 0,07678)		V = 0,1258 (p = 0,04151*)	
Q1 (n = 447)		87,25 % (390)		26,62 % (119)		52,94 % (63)
Q2 (n = 447)		88,79 % (396)		22,20 % (99)		58,59 % (58)
Q3 (n = 447)		87,67 % (391)		18,83 % (84)		44,05 % (37)
Q4 (n = 447)		89,71 % (401)		25,06 % (112)		44,64 % (64)
Gender (n = 1797)	V = 0,04117 (p = 0,5502)		V = 0,02905 (p = 0,4686)		V = 0,06697 (p = 0,3935)	
Men (n = 950)		87,69 % (833)		24,00 % (228)		50,00 % (114)
Women (847)		89,14 % (755)		22,20 % (188)		50,00 % (94)
Education (n = 1799)	V = 0,08455 (p = 0,0001*)		V = 0,1291 (p = 3,846e-09*)		V = 0,1475 (p = 0,05333)	
University (n = 134)		96,27 % (129)		38,81 % (52)		65,38 % (34)
UAS (n = 462)		93,72 % (433)		32,03 % (148)		55,41 % (82)
Vocational (n = 602)		87,71 % (528)		17,61 % (106)		46,23 % (49)
Secondary (n = 429)		84,85 % (364)		17,72 % (76)		43,42 % (33)
Primary (n = 132)		78,04 % (68)		21,97 % (29)		31,03 % (9)
Other (n = 38)		81,58 % (31)		12,16 % (5)		20,00 % (1)
Cancer type (n = 1840)	V = 0,086 (p = 0,2428)		V = 0,08695 (p = 0,2677)		V = 0,2034 (p = 0,066)	
Breast (n = 519)		89,79 % (466)		23,51 % (122)		54,10 % (66)
Prostate (n = 464)		89,66 % (416)		25,86 % (120)		50,00 % (60)
Head-neck (n = 215)		87,44 % (188)		28,37 % (61)		63,93 % (39)
Lung (n = 196)		89,28 % (175)		18,37 % (36)		50,00 % (18)
Gastrointestinal (n = 125)		84,00 % (105)		16,00 % (20)		25,00 % (5)
Haematological (n = 75)		89,34 % (67)		18,67 % (14)		28,57 % (4)
Neurological (n = 64)		84,37 % (54)		26,56 % (17)		64,71 % (11)
Gynaecological (n = 52)		86,54 % (45)		17,31 % (9)		33,33 % (3)
Bladder (n = 35)		94,29 % (33)		22,86 % (8)		50,00 % (4)
Skin (n = 24)		83,34 % (20)		33,33 % (8)		12,50 % (1)
Renal (n = 8)		75,00 % (6)		12,50 % (1)		00,00 % (0)
Other (n = 49)		79,59 % (39)		14,29 % (7)		42,86 % (3)
Non-cancer (n = 14)		78,57 % (11)		14,29 (2)		00,00 % (0)

a : The percentage of patients experiencing multiple options was derived from the number of patients experiencing multiple options divided by the number of patients experiencing a decision.

Age was associated with experiencing multiple treatment options. In quartile 1 (27,0 to 60,25 years, 52,9%, n = 63) and 2 (60,25 to 68 years, 59,6%, n = 58), patients were more likely to experience multiple options than in quartile 3 (68 to 75 years, 44,1%, n = 37) and 4 (75 to 92 years, 44,6%, n = 50) (Cramér’s V = 0,1258, p = 0,0415). Although the result was not significant for disease type (p = 0,066), the proportion of patients experiencing multiple options ranged from 12,5% (n = 1) in skin cancer to 63,93% (n = 39) in head-neck and 64,71% (n = 11) in neurological cancer. Associations of gender, treatment year and educational level with number of options were also not significant.

## Discussion

This study assessed experienced SDM during RT intakes and patients’ preferences regarding SDM. In this section, we will contextualise the study and its findings, beginning with patient preferences in RT, followed with their experiences and concluding with suggestions for implementation and study limitations.

### SDM preferences

Our study affirms a strong preference for SDM among RT patients, aligning with prior oncology research in the Netherlands. Kuijpers and colleagues found an 88,1% preference for SDM in cancer patients nationwide, akin to our 88,3% finding [15]. SDM preference is evident in preference-sensitive RT decisions, such as adjuvant treatment choices for women with breast cancer [31] and decisions on surgery or stereotactic ablative RT for early-stage non-small cell lung cancer [32]. Our study extends the relevance of SDM preferences in oncology to the entire RT context.

Moreover, our results highlight a link between a higher educational level and SDM preference. Evidence in literature about the relationship between educational level and SDM preference is mixed, with international studies reporting negative and null effects [33], [34], while others show a positive correlation [35], [36]. The observed negative relationship in the international study may have occurred due to the inclusion of large samples with a non-Western culture, where norms surrounding interactions with health-care providers might be different than in our RT centre.

### SDM experiences

Despite patients expressing a clear preference for SDM in RT, our study uncovered a notable disparity between preferences and experiences. 23,1% of patients in our study reporting experiencing a choice to be made during their intake, by either the physician or themselves. In line with a recent review, we did not find any correlation between cancer type and experienced SDM, supporting the generalisability of our findings. However, our study did not assess the severity of the cancer, for some studies suggest that this might be associated with SDM occurrence [37]. A Dutch study on breast cancer patients’ perception of choice demonstrated similar results with regards to experienced SDM: 32 % of patients undergoing adjuvant chemotherapy and 14% of patients not undergoing chemotherapy experienced a treatment choice [38]. Yet, experiencing a choice does not inherently imply that SDM occurred, and our study lacked specific SDM measurement tools like SDM-Q-9, iSHARE, or collaboRATE. However, it could be inferred that patients who did not encounter decisions or multiple treatment options, did not experience SDM, as SDM requires patients to be aware of a decision and options associated with that decision [16], [39], [40]. This constitutes to a maximum of 11,6% of our sample experiencing SDM. In comparison, without any SDM interventions, a Dutch study focusing on pre-operative RT decisions in rectal cancer revealed that patient values and or treatment preferences were discussed in 54% of consultations [41], and 56 % of high-intermediate risk endometrial cancer patients undergoing vaginal vault brachytherapy perceived a decision about their treatment [42]. Future studies could aim to assess how often SDM occurs in RT using physician perspectives or observational measurements.

Although our survey incorporated the scientific definition of SDM, including consideration of “all possible treatments,” half of the patients reporting experiencing that a treatment decision had to be made, did not report having more than one treatment option. Qualitative insights suggest that patients may perceive a decision as shared if they feel engaged and arrive at the same conclusion, for example when asked for consent [31], [43], [44]. Furthermore, patients might not recognise passive options like watchful waiting or non-treatment as true treatment options, potentially explaining why half of our patients who experienced a choice did not report having multiple options [45]. Thus, when using patient perspectives on SDM, it is recommended to understand what patients consider SDM to enable comparison between studies. However, even when omitting the requirement for multiple treatment options, it can still be concluded that there is room for improvement regarding perceived SDM.

When investigating the role of patient characteristics in their experience with SDM, a lower educational level was associated with less experienced SDM in RT. A secondary analysis of American trials using the OPTION-12 instrument replicated that education is associated with experienced SDM, as their control-group patients with the lowest education scored 6,5 points lower than those with the highest education [46]. Patients with a lower education are much more likely to have limited health literacy (LHL) [47]. These patients are prone to experience problems with *obtaining* health information, *communicating* about health, and *applying* health information [48], which are critical for meaningful engagement with healthcare professionals and experiencing SDM [49]. While our study did not measure health literacy, it is conceivable that the positive association between education and experienced SDM is affected by health literacy.

### Recommendations towards improved SDM implementation

This study not only identifies challenges but also highlights potential solutions for implementing SDM in RT by assessing barriers to SDM and rating of potential solutions. Within our sample, patients often communicated a desire for clearer patient-physician communication, which could be addressed by training radiation oncologists. Potentially useful components of a training might be the modelling of good SDM practices, for example by peers or actors [50], learning *which* information patients want to have, and *how* to communicate this to patients with LHL. Breast cancer patients facing an RT decision mostly valued information about risk of recurrence with and without RT, their individualised risk of side-effects, and the impact of side-effects on quality of life [51], [52]. A systematic review on risk communication strategies for patients with LHL recommends complementing verbal risk communication with numerical and/or visual risk communication, preferably using natural frequencies instead of percentages, and using icon arrays [53]. Furthermore, almost one in three patients experiencing that a decision had to be made, reported being unaware of their ability to participate in decision-making. Given the likelihood that this number may be higher among those not experiencing a decision, SDM implementation efforts could benefit from raising SDM awareness among RT patients. The “Ask Three Questions” approach, equipping patients with crucial questions, presents a practical strategy. This approach can be delivered through diverse media, including a waiting room video, and has been implemented and evaluated with positive results [54], [55], [56], [57]. Lastly, assistance in weighing benefits and drawbacks of treatment options were frequently selected solutions to improve SDM. Tailored decision aids specific to their options could prove beneficial, enhancing patient satisfaction, knowledge, perceived control, and SDM in RT [24].

While acknowledging that efforts should be made to implement SDM, it may not be feasible or desirable to perform SDM in all RT intake consultations. A recent systematic review indicated that SDM was generally deemed appropriate in case of preference-sensitive options, multiple options, high-impact decisions and situations of uncertainty, and deemed inappropriate in situations without equipoise or requiring immediate life-saving actions. However, perceptions in literature of appropriateness of SDM were conflicting in cases of having a singular best treatment option, decisions with low impact and short timeframes [58]. Thus, identifying and communicating which specific decisional situations are *always* suitable for SDM in radiation oncologists’ clinical practice might serve as a starting point for improve SDM utilization. Further research should focus on experimenting with SDM in situations with one best option, low-impact decisions and short time-frame decisions, and assessing the effect thereof using patient-reported outcomes.

### Strengths and limitations

This study faced limitations, including the absence of data on satisfaction among patients not experiencing a decision, recall issues, and risk of selection bias. Firstly, to manage participant burden, patients without a decision did not answer questions on decision effects, reducing the sample size for satisfaction-related items and hindering comparisons and generalization. Secondly, some participants had intake consultations > 1 year ago, raising recall concerns about decision-making processes and proposed treatment options. To address this, we used more general questions than detailed items in validated instruments measuring SDM. Furthermore, we did not find any associations between SDM preference, experience or number of options and treatment year. Thirdly, as with all survey studies, there was a risk of selection bias, as the population of patients willing to answer a survey might differ from the entire population of RT patients. Despite these limitations, the study had strengths, including a large and diverse sample representing various educational levels and cancer types which was representative of the region, enhancing external validity [59], [60], [61]. Additionally, incorporating patients who did not perceive an option allowed for a comprehensive exploration of SDM experiences across the entire RT patient spectrum.

## Conclusion

The identified disparity between experienced and preferred decision-making in this RT centre echoes earlier findings of insufficient SDM in oncology, extending these concerns to the context of RT. To bridge this gap, concentrated efforts are imperative to integrate SDM into RT, recognizing the need for tailored strategies adapted to its specific context. This may involve targeting patient groups with lower educational levels and training radiation oncologists. Addressing these challenges is pivotal for improving patient outcomes and satisfaction in RT.

## Declaration of competing interest

The authors declare that they have no known competing financial interests or personal relationships that could have appeared to influence the work reported in this paper.
