# Corrigendum to “Bridging the care gap: patients’ needs and experiences regarding shared decision-making in radiotherapy” [Clin. Translat. Radiat. Oncol. (2025) 100897]

**DOI:** 10.1016/j.ctro.2024.100903

**Published:** 2024-12-20

**Authors:** A.R. van Hienen, C.J.W. Offermann, L.J. Boersma, M.J.G. Jacobs, R.R.R. Fijten

**Affiliations:** a Department of Radiation Oncology (MAASTRO), GROW Research Institute for Oncology and Reproduction, Maastricht University Medical Centre, Maastricht, Dr Tanslaan 12, 6229 ET, the Netherlands; b Maastro Clinic, Research Affairs department, Maastricht, Dr Tanslaan 12, 6229 ET, The Netherlands; c Department of Management, Tilburg School of Economics and Management, Tilburg University, Tilburg, Warandelaan 2, 5037 AB, The Netherlands

The authors regret that in the highlights section of the online article, it states that “23,1 % of patients did not experience a choice during their RT intake.”. This should be “23,1 % of patients experienced a choice during their RT intake.”, as mentioned throughout the remainder of the article.

The authors would like to apologise for any inconvenience caused.

