# Supplementary material for: Bridging the care gap: patients’ needs and experiences regarding shared decision-making in radiotherapy
Source: Clin Transl Radiat Oncol. 2024 Nov 24;50:100897. doi: 10.1016/j.ctro.2024.100897 (PMC11636202; doi:10.1016/j.ctro.2024.100897)
Supplement: Supplementary Data 1 [file mmc1.docx]

1. *[CORRESPONDING AUTHOR] A.R. van Hienen ^a^ (*[*anniek.vanhienen@maastro.nl*](mailto:anniek.vanhienen@maastro.nl)*;* [*https://orcid.org/0009-0000-9454-6124*](https://orcid.org/0009-0000-9454-6124) *)*
2. *C.J.W. Offermann^b^ (*[*claudia.offermann@maastro.nl*](mailto:claudia.offermann@maastro.nl)*)*
3. *L.J. Boersma^a^ ([liesbeth.boersma@maastro.nl](mailto:liesbeth.boersma@maastro.nl); <https://orcid.org/0000-0001-9173-279X> )*
4. *M.J.G. Jacobs^c^ (*[*m.j.g.jacobs@tilburguniversity.edu*](mailto:m.j.g.jacobs@tilburguniversity.edu)*;* [*https://orcid.org/0000-0001-8832-2378*](https://orcid.org/0000-0001-8832-2378) *)*

*R.R.R. Fijten^a^ (*[*rianne.fijten@maastro.nl*](mailto:rianne.fijten@maastro.nl)*;* [*https://orcid.org/0000-0002-1964-6317*](https://orcid.org/0000-0002-1964-6317) *)*
